# Intracardiac thrombus formations despite continuous oral anticoagulation in atrial fibrillation patients undergoing catheter ablation procedures: pilot development of a machine learning prediction model

**DOI:** 10.3389/fcvm.2026.1707003

**Published:** 2026-06-04

**Authors:** F. Ratajczak, F. Hohendanner, S. Haack, L. Boldt, A. S. Parwani, F. Blaschke, M. Schneider-Reigbert, F. Spinka, M. Bock, A. Meyer, E. Heil, G. Hindricks, D. Schoeppenthau

**Affiliations:** 1 Charité—Universitätsmedizin Berlin, Corporate Member of Freie Universität Berlin and Humboldt-Universität zu Berlin, Berlin, Germany; 2 Deutsches Herzzentrum der Charité, Klinik, Department of Cardiology, Angiology and Intensive Care Medicine, Berlin, Germany; 3 DZHK (German Centre for Cardiovascular Research), Berlin, German Charité; 4 Deutsches Herzzentrum der Charité, Department of Cardiology, Angiology and Intensive Care Medicine, Berlin, Germany; 5 Deutsches Herzzentrum der Charité, Department of Cardiothoracic and Vascular Surgery, Berlin, Germany; 6 Berlin Institute for the Foundations of Learning and Data (BIFOLD), Technical University of Berlin, Berlin, Germany

**Keywords:** atrial fibrillation, catheter ablation, left atrial thrombus, machine learning, oral anticoagulation

## Abstract

**Background:**

Atrial fibrillation (AF) increases the risk of stroke, primarily due to thrombus formations (TF) in the left atrium (LA), especially in advanced atrial disease. Undetected TF despite continuous oral anticoagulation remain a safety concern, particularly in patients undergoing AF catheter ablations (CA) procedures. This retrospective study aimed to develop a machine learning (ML) model to predict TF in those patients.

**Methods:**

A retrospective cohort of 1335 AF patients who underwent CA and transesophageal echocardiography were screened for TF. Initially, *n* = 93 TF+ patients were identified and propensity-score-matched (age, sex) to 93 TF− controls. Clinical, echocardiographic data and p-wave morphology in sinus rhythm (SR) were assessed. After excluding patients without SR ECGs 65 TF+ and 84 TF− patients were used for the ML models. External validation was performed.

**Results:**

We analyzed 149 patients undergoing LA CA procedures on DOACs 96.0%/VKA 4.0%. The TF+ patients (20% solid thrombi, 80% prethrombotic formations) had a mean CHA₂DS₂-VASc of 3.89 and were in clinical arrhythmias in 96.9% at the time of TF, seen in a TOE performed before planned CA. The ML models showed a good performance in recognizing TF in the test-dataset, achieving a ROC AUC of 0.886 and 0.826 which outperformed CHA₂DS₂-VASc in our cohort (AUC 0.641). Some P-wave-characteristics in SR are ranked among the top ML features.

**Conclusion:**

ML algorithms, including echo parameters and p-Wave parameters reflecting atrial substrate, offer a promising approach for accurate prediction of TF in anticoagulated AF patients undergoing CA. Further validation could enable implementation of a clinically applicable risk stratification tool to enhance preprocedural decision-making.

## 1 Introduction

Atrial fibrillation (AF) generates a risk for thromboembolic events, particularly ischemic stroke due to thrombus formation (TF) in the left atrium (LA) and in the left atrial appendage (LAA) (1).

While guideline-directed oral anticoagulation (OAC) significantly reduces thromboembolic risk, thrombi may still be observed despite adequate anticoagulation, particularly in the context of advanced atrial cardiomyopathy (ACM) (2, 3). This represents a clinically meaningful problem in a catheter ablation setting, particularly with regard to identifying which patients need to be screened for TF.

Since the presence of atrial thrombus is a contraindication for catheter ablation due to associated risk of procedural thromboembolic complications transesophageal echocardiography (TOE) may be performed to exclude TF. Current guidelines do not mandate TOE for all patients on uninterrupted oral anticoagulation (2, 4). Identifying patients at high risk of TF despite OAC may help guide selective use of TOE while avoiding missed thrombi.

Several studies have attempted to characterize predictors of TF. Clinical risk scores such as CHA₂DS_2_-VASc or CHA_2_DS_2_-VA, though widely used for stroke risk stratification, have limited efficacy for actual thrombus detection in anticoagulated patients (5). Other parameters have been associated with thrombus or prethrombotic formations but are rarely used for individualized prediction (6). Likewise, electrocardiogram (ECG) markers of atrial remodeling, such as prolonged P-wave duration or advanced interatrial block, have been linked to atrial thrombogenesis but remain underutilized in risk models (7).

Recent advances in machine learning (ML) have enabled the integration of multidimensional clinical, echocardiographic, and ECG data to improve risk prediction in cardiovascular medicine. Previous studies have explored the use of ML to predict TF or stroke risk in patients with AF, but electrocardiographic parameters in SR have not yet been used as main features (8–11).

This retrospective study aims to develop a machine learning model to predict TF in AF patients undergoing LA catheter ablations (CA) under OAC by analyzing clinical data, echo characteristics and parameters of patient's SR ECGs as potential markers of atrial remodeling and thrombogenic substrate.

We explored whether a ML model could be trained to differentiate between patients with and without TF, using these multimodal features as input.

## 2 Method

### 2.1 Study population

All patients included in this study were treated at a tertiary academic medical center. We retrospectively identified adult patients with AF who were referred for LA catheter ablation (*de novo* or re-do procedures for AF or atrial tachycardia) between June 2019 and December 2024. Patients were excluded if digital access to echocardiographic records was incomplete or if TF was documented without concurrent OAC.

Two subsets were constructed by first identifying a total of 93 patients with confirmed LA thrombus or prethrombotic formation (TF+) through an automated query using relevant ICD codes and keyword search (e.g., “thrombus”, “sludge”, “pre-thrombus-formation”) in diagnosis fields and free-text TOE reports. All cases were manually reviewed to confirm the presence of thrombus. Then the control group (TF−) was selected from the same pool of ablation-referred patients but had an exclusion of LA thrombi in a TOE. Because age is a major determinant of thromboembolic risk in AF and sex is a well-recognized risk modifier, we used propensity score matching as a design-stage approach to create balanced comparison groups, thereby reducing confounding by these factors and enabling a clearer assessment of incremental predictive features associated with TF status. We thereby aimed to create balanced groups that would allow us to identify additional predictive features beyond these established risk factors and consider this approach a conservative strategy to reduce confounding. Matching was performed in a 1:1 ratio based on age and sex using propensity score matching. This was implemented in R version 2024.04.2+76413 (12) using the MatchIt package (13) (version 4.1.0) with nearest neighbor matching and a caliper of 0.25. Propensity scores were estimated via logistic regression (method = “nearest”, distance = “glm”).

**Figure 1:**
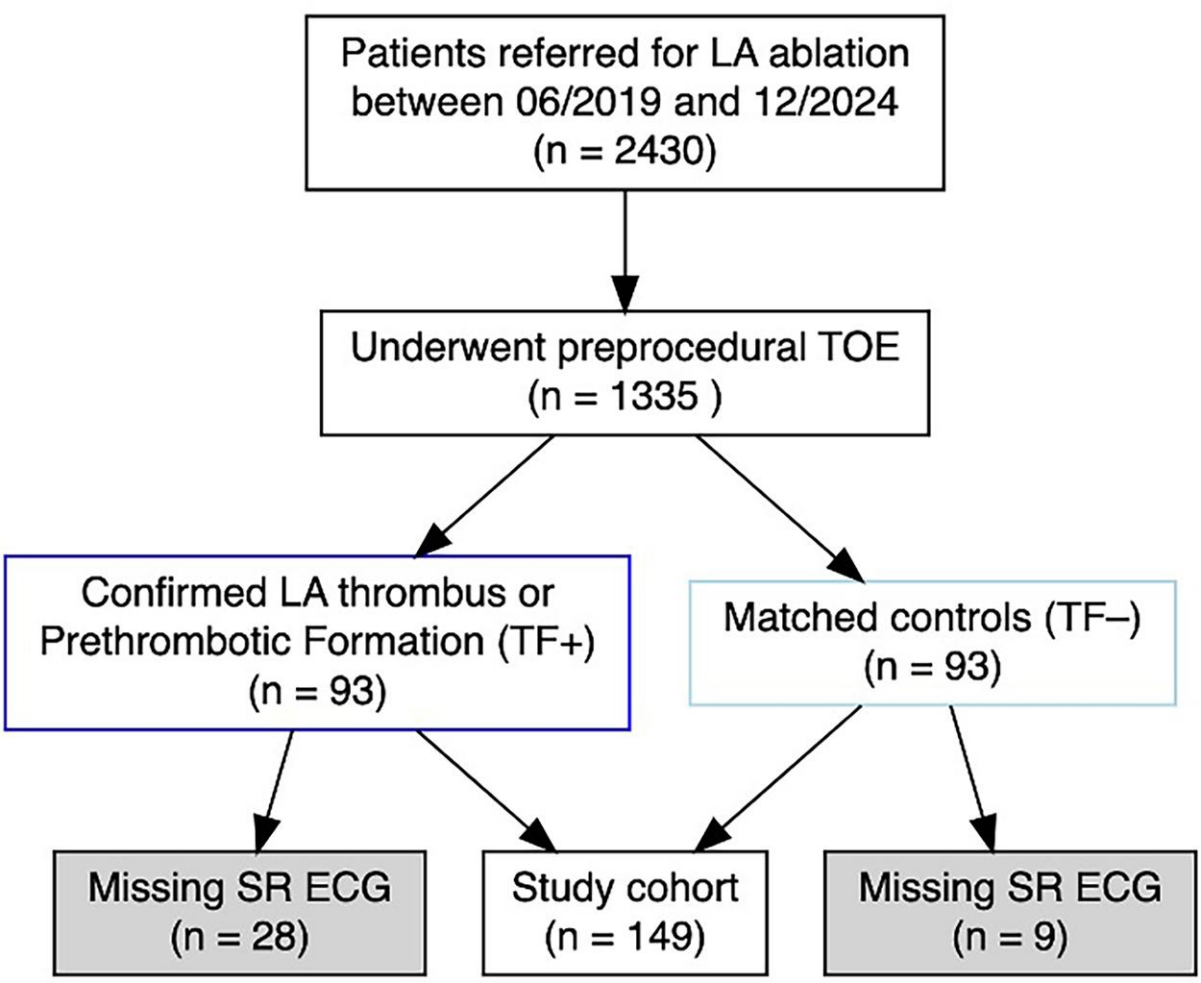
Flow chart demonstrating patient selection for analysis.

After matching, both groups were screened for the availability and quality of baseline 12-lead ECGs in the clinical records within one year prior to thrombus detection or CA. Since the relevance of P-wave morphology parameters as a marker of atrial substrate, patients were excluded if no SR ECG was available or if the ECG was of insufficient technical quality due to noise or artifacts. After this final step, 65 TF+ and 84 TF− patients remained for analysis (see Figure 1). Clinical data, echocardiographic parameters, and ECG features were manually extracted from electronic medical records.

### 2.2 Echocardiographic assessment

Echocardiographic examinations were performed by an experienced echocardiographer using commercially available equipment (Vivid E95, Vivid7, GE Healthcare, Chicago, IL, USA, 6Vt-D 4D Multiplane Transesophageal Probe). Echocardiography was performed in accordance with the recommendations of the American Society of Echocardiography and the European Association of Cardiovascular Imaging (14). Thrombus was defined as an organized, well-defined mass. Prethrombotic formations were defined as persistent swirling echodensity throughout the cardiac cycle exhibiting a viscid appearance (termed sludge) in the absence of a discrete mass using with appropriate gain settings to minimize the risk of artifact (15). During the study period our institutional protocol mandated routine pre-procedural TOE for all consecutive patients referred for left atrial ablation irrespective of clinical risk profile or anticoagulation regimen. The decision to perform TOE was not left to clinician discretion.

### 2.3 ECG parameters

We analyzed the present rhythm before transesophageal (TOE) imaging (AF, SR or atrial tachycardia). For the measurement of P-wave parameters all available SR ECG's in the clinical records were identified. In many patients, SR ECGs were available from earlier phases of their clinical course. If multiple SR ECGs were available, we selected the recording closest to the TF diagnosis (in the TF group) or to the catheter ablation procedure (in the control group). Standard ECG parameters (heart rate, QRS duration, p and R electrical axis, QT duration) were taken from automated machine readouts. P-wave morphology parameters were measured manually by two independent observers. The following parameters were selected: P-wave duration (lead II and V1), P-wave amplitude, biphasic and bifid morphology in II, biphasic or negative morphology in V1, P-terminal force in V1 (PTFV1), P-wave dispersion, interatrial block). Normal values were based on definitions from the current clinical consensus statement on ACM (7).

### 2.4 Statistical analysis

Descriptive statistics were performed using R version 2024.04.2+764(12) with the tableone package (version 0.13.2) (16). The CreateTableOne() function with the strata argument was used to compare baseline characteristics between groups. Student's *t*-test for continuous variables and the chi-squared test for categorical variables were used. Continuous variables are reported as means and standard deviation, and categorical variables as absolute numbers and percentages.

### 2.5 Machine learning

Three binary classification models were developed using the packages xgboost (17), ranger (18) and lightgbm (19) in R version 2024.04.2+764 (12).

The dataset was randomly divided into a training set (80%) and a test set (20%) using the createDataPartition() function from the caret package (20), with a fixed seed to ensure reproducibility.

For the XGBoost model, hyperparameters were set to maximum tree depth of 3, a learning rate (eta) of 0.01, and subsampling rates of 0.8 for both rows and features. Regularization was applied through L1 (alpha = 0.5) and L2 (lambda = 1) penalties, and a minimum loss reduction (gamma = 1) was required for additional tree splits.

Model performance was evaluated on the independent test set. Predicted probabilities were converted to binary outcomes using a threshold of 0.4. Discrimination was quantified using ROC-AUC (21) and precision-recall AUC (22), and classification quality was further assessed by accuracy, precision, recall, F1 score, and confusion matrices (20).

The Random Forest classifier was trained using 1,000 trees, with the number of variables randomly selected at each split (mtry) set to the square root of the total number of features. Class probabilities were used to evaluate ROC-AUC and PR-AUC, and binary predictions were made using a threshold of 0.5. Performance metrics were calculated identically to those of the XGBoost model.

The LightGBM model employed histogram-based boosting with gradient descent optimization. Key parameters included 10 leaves per tree, a learning rate of 0.05, and subsampling fractions of 0.8 for features and 0.9 for rows. The model was trained for up to 1,000 boosting rounds with performance monitored on the training data. Predictions on the test set were thresholded at 0.2 to optimize sensitivity. As with the other models, LightGBM performance was assessed using ROC-AUC, PR-AUC, and standard classification metrics.

All three models were evaluated under identical conditions using the same training and testing splits, allowing for a direct comparison of predictive performance in identifying patients at risk for treatment failure. For xgboost and LightGBM a 5-fold cross validation was additionally performed on the training set to ensure robustness and validate consistency in discriminative performance (17, 19).

To determine the optimal probability threshold for binary classification, Youden's J statistic was calculated from the receiver operating characteristic (ROC) curve separately for each classifier. The ROC curve was generated using the predicted probabilities, and the true positive rate (TPR) and false positive rate (FPR) were extracted for all threshold values. Youden's J statistic was defined as J = TPR−FPR, and the threshold yielding the maximum J was selected as the optimal cut-off. This method ensures an optimal balance between sensitivity and specificity, independent of disease prevalence. The resulting threshold was used for final classification and performance evaluation of the model.

To assess the contribution of individual input features to the ML models, feature importance was calculated separately for each algorithm using established model-specific methods. For the XGBoost model, feature importance was computed using the xgb.importance() function from the xgboost package (17), using gain-based importance as the primary metric of interest. Similarly, for the LightGBM model, we extracted gain-based feature importance using the lgb.importance() function from the lightgbm package (19).

For the Random Forest model trained with the ranger package, impurity-based importance values were extracted directly from the trained model object (ranger$variable.importance) (18).

### 2.6 Shap analysis

For all three classifiers, model interpretability was assessed by values and permutation feature importance. SHAP values were obtained using the model-specific implementations: TreeSHAP [predict(…, predcontrib = TRUE)] for XGBoost, TreeSHAP (predict(…, type = “contrib”) for LightGBM, and Monte-Carlo Shapley approximation [fastshap package (23)] for the Random Forest. All SHAP outputs were processed and visualized with the shapviz package (24).

To quantify robustness, we performed 5-fold cross-validation using the caret package. For each fold, mean absolute SHAP values were computed, and stability of the resulting feature rankings was evaluated via Spearman rank correlations and dispersion across folds (interquartile ranges and boxplots). In addition, bootstrap resampling (B = 1,000) was applied to derive 95% confidence intervals for global mean absolute SHAP values.

Permutation feature importance was computed with the vip package (25), using ΔAUC as the evaluation metric. Confidence intervals were obtained from repeated permutations, and results were visualized as forest plots.

### 2.7 External validation

For external validation, 16 patients from a separate tertiary academic medical center who underwent CA for AF in 2024 were included (2 TF+/14 TF−). Thrombus-positive cases were identified using ICD codes and confirmed by manual review. Thrombus-negative patients were randomly selected from those with documented thrombus exclusion on TOE. Only patients with available SR ECG and digital echo data were included.

## 3 Results

### 3.1 Baseline characteristics

A total of 149 patients were included in the analysis, with 65 patients in the TF group and 84 in the control group. Baseline characteristics are summarized in Table 1. Age and sex were similar between groups due to prior propensity score matching.

**Table 1:** Clinical baseline characteristics of the training group comparing TF+ and TF− group.

Characteristic	All	TF−	TF+	*p*
*n*	149	84	65	
BMI [mean (SD)]	27.64 (5.09)	27.10 (4.72)	28.32 (5.49)	0.151
Persistent AF [*n* (%)]	106 (71.1)	49 (58.3)	57 (87.7)	<0.001
Sex = male [*n* (%)]	65 (43.6)	37 (44.0)	28 (43.1)	1.000
Structural Heart Disease [*n* (%)]	39 (26.2)	11 (13.1)	28 (43.1)	<0.001
Renal Insufficiency [*n* (%)]	37 (24.8)	14 (16.7)	23 (35.4)	0.015
Congestive Heart Failure [*n* (%)]	66 (44.3)	28 (33.3)	38 (58.5)	0.004
Hypertension [*n* (%)]	125 (83.9)	64 (76.2)	61 (93.8)	0.007
Diabetes [*n* (%)]	32 (21.5)	12 (14.3)	20 (30.8)	0.026
TIA [*n* (%)]	3 (2.0)	2 (2.4)	1 (1.5)	1.000
Stroke [*n* (%)]	20 (13.4)	12 (14.3)	8 (12.3)	0.913
Other Thromboembolic Events [*n* (%)]	2 (1.3)	1 (1.2)	1 (1.5)	1.000
Vascular Disease [*n* (%)]	59 (39.6)	27 (32.1)	32 (49.2)	0.052
Age [mean (SD)]	71.70 (8.48)	72.14 (8.74)	71.14 (8.16)	0.475
CHA₂DS₂-VASc Score [mean (SD)]	3.50 (1.48)	3.19 (1.59)	3.89 (1.23)	0.004
CHA₂DS₂-VASc Score [*n* (%)]				0.022
0	1 (0.7)	1 (1.2)	0 (0.0)	
1	16 (10.7)	14 (16.7)	2 (3.1)	
2	21 (14.1)	14 (16.7)	7 (10.8)	
3	35 (23.5)	20 (23.8)	15 (23.1)	
4	35 (23.5)	18 (21.4)	17 (26.2)	
5	30 (20.1)	10 (11.9)	20 (30.8)	
6	9 (6.0)	5 (6.0)	4 (6.2)	
7	2 (1.3)	2 (2.4)	0 (0.0)	
Creatinine, mg/dL [mean (SD)]	1.05 (0.37)	0.99 (0.26)	1.12 (0.48)	0.034
eGFR, mL/min/1.73 m^2^ [mean (SD)]	66.55 (17.13)	68.42 (15.47)	64.06 (18.97)	0.128
LAVI, mL/m^2^ [mean (SD)]	45.24 (14.46)	40.81 (13.09)	51.32 (14.13)	<0.001
LVEF, % [mean (SD)]	54.25 (10.67)	56.74 (9.44)	50.95 (11.36)	0.001
Septum Thickness, ED, mm [mean (SD)]	11.40 (1.97)	10.94 (1.84)	12.04 (1.98)	0.001
Posterior Wall, ED, mm [mean (SD)]	10.02 (1.83)	9.86 (1.83)	10.27 (1.82)	0.233

TF, thrombus formation; BMI, body mass index; AF, atrial fibrillation; TIA, transient ischemic attack; eGFR, glomerular filtration rate (CKD-EPI calculation); LAVI, left atrial volume index; LVEF, left ventricular ejection fraction; ED, end-diastole.

In the comparative analysis of baseline characteristics, patients with thrombus formation (TF) demonstrated a significantly higher prevalence of persistent AF (87.7% vs. 58.3%, *p* < 0.001), structural heart disease (43.1% vs. 13.1%, *p* < 0.001), renal insufficiency (35.4% vs. 16.7%, *p* = 0.015), congestive heart failure (58.5% vs. 33.3%, *p* = 0.004), hypertension (93.8% vs. 76.2%, *p* = 0.007), and diabetes (30.8% vs. 14.3%, *p* = 0.026) compared to the group without TF. Additionally, patients in the TF group had higher CHA₂DS_2_-VA and CHA_2_DS_2_-VASc Scores (*p* = 0.005 and *p* = 0.022, respectively).

**Figure 2:**
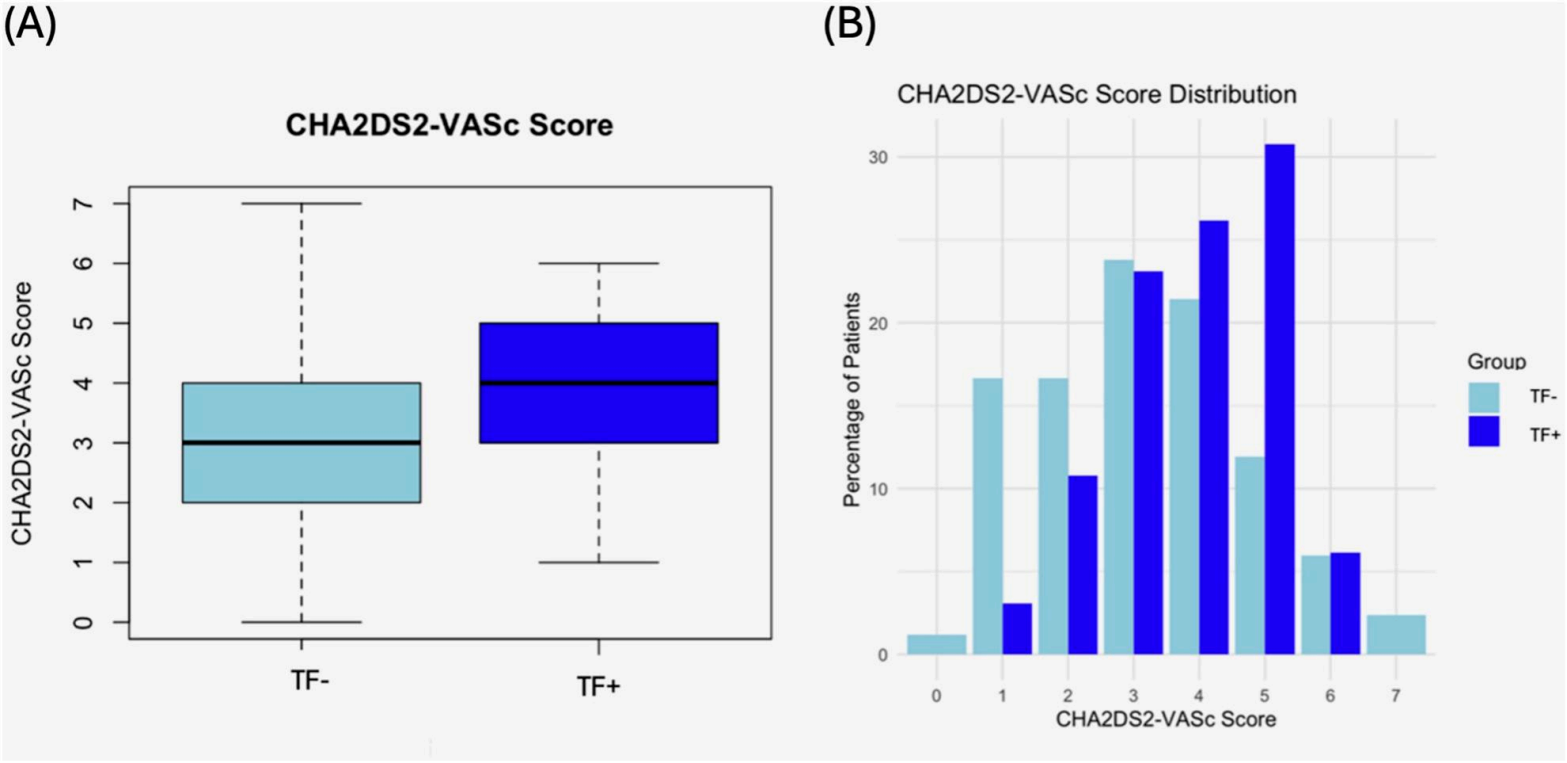
Clinical CHA_2_DS_2_-VASc scoring in the TF+ and TF− group; (**A**) Boxplot of the CHA_2_DS_2_-VASc in each group; (**B**) CHA2DS2-VASc score distribution in each group.

The distribution of CHA_2_DS_2_-VASc Scores in both groups is illustrated in Figure 2.

Furthermore, patients in the TF group showed elevated creatinine levels (1.12 ± 0.48 vs. 0.99 ± 0.26 mg/dL, *p* = 0.034), larger left atrial volume index (51.32 ± 14.13 vs. 40.81 ± 13.09 mL/m^2^, *p* < 0.001), and reduced left ventricular ejection fraction (50.95 ± 11.36% vs. 56.74 ± 9.44%, *p* = 0.001).

### 3.2 Oral anticoagulation

All patients in the study were on continuous OAC at the time of thrombus detection or exclusion. Details can be found in Table 2. The majority received direct oral anticoagulants (DOACs), with Apixaban being the most frequently prescribed agent (47.0%), followed by Rivaroxaban (34.2%), Edoxaban (11.4%), and Dabigatran (3.4%). Only a small proportion of patients (4.0%) were treated with a vitamin K antagonist (VKA; Phenprocoumon). The distribution of anticoagulant types did not significantly differ between the two groups (*p* = 0.089).

**Table 2:** OAC status at the time of first documented TF or thrombus exclusion (%).

Oral Anticoagulant	All	TF−	TF+	*P*
*n*	149	84	65	0.089
Apixaban [*n* (%)]	70 (47.0)	40 (47.6)	30 (46.2)	
Dabigatran [*n* (%)]	5 (3.4)	1 (1.2)	4 (6.2)	
Edoxaban [*n* (%)]	17 (11.4)	9 (10.7)	8 (12.3)	
Phenprocoumon [*n* (%)]	6 (4.0)	1 (1.2)	5 (7.7)	
Rivaroxaban [*n* (%)]	51 (34.2)	33 (39.3)	18 (27.7)	

### 3.3 ECG measurements

In our cohort, 96.9% of TF+ patients were in an atrial arrhythmia at the time of TOE (AF in 89.2%, atrial tachycardia in 4.7%, and typical atrial flutter in 1.5%). The remaining 3.1% (*n* = 2) were in SR but had undergone electrical cardioversion within the week preceding thrombus detection. SR ECG characteristics of the two groups are presented in Table 3. The TF+ group exhibited significantly higher P-wave dispersion (45.31 ± 23.05 ms vs. 32.86 ± 19.19 ms, *p* < 0.001) and a higher prevalence of abnormal P-wave dispersion (63.1% vs. 39.3%, *p* = 0.007). Biphasic P-waves in lead II were also more frequent in the TF+ group (24.6% vs. 10.8%, *p* = 0.046), as were abnormal P-wave durations in lead II (58.5% vs. 39.8%, *p* = 0.036). In contrast, QRS duration, QTc interval, LBBB prevalence, PQ time, P-wave duration and morphology in lead V1, and the presence of advanced interatrial block did not differ significantly between groups.

**Table 3:** Sinus rhythm ECG characteristics compared between TF− group and TF+ group.

Characteristic	TF−	TF+	p
*n*	84	65	
Heart Rate, bpm [mean (SD)]	67.43 (11.30)	66.43 (11.42)	0.596
PQ Time, ms [mean (SD)]	186.80 (36.74)	193.08 (39.85)	0.322
QRS Duration, ms [mean (SD)]	98.92 (19.66)	104.25 (21.01)	0.113
QT Time, ms [mean (SD)]	416.83 (38.49)	426.63 (43.32)	0.147
QTc, ms [mean (SD)]	434.19 (37.68)	445.28 (33.63)	0.064
AV Block 1 [*n* (%)]	25 (29.8)	22 (33.8)	0.723
Bundle Branch Block [*n* (%)]			0.459
None	69 (82.1)	48 (73.8)	
LBBB	5 (6.0)	8 (12.3)	
RBBB	6 (7.1)	4 (6.2)	
Incomplete RBBB	4 (4.8)	5 (7.7)	
P Axis, degree [mean (SD)]	60.49 (21.25)	56.51 (26.33)	0.310
Abnormal P Wave Axis [*n* (%)]	21 (25.0)	15 (23.1)	0.937
R Axis, degree [mean (SD)]	29.33 (51.27)	24.54 (45.93)	0.555
Abnormal R Axis [*n* (%)]	15 (17.9)	14 (21.5)	0.723
P Wave Duration in II, ms [mean (SD)]	122.83 (25.87)	131.54 (29.63)	0.059
Abnormal P Wave Duration in II (*n* (%)	33 (39.8)	38 (58.5)	0.036
Biphasic P Wave in II [*n* (%)]	9 (10.8)	16 (24.6)	0.046
Bifid P Wave in II [*n* (%)]	30 (36.1)	15 (23.1)	0.125
Abnormal Morphology II [*n* (%)]	39 (47.0)	31 (47.7)	1.000
P Wave Amplitude in II, mV [mean (SD)]	0.14 (0.05)	0.12 (0.05)	0.109
Abnormal P Wave Amplitude in II [*n* (%)]	2 (2.4)	1 (1.5)	1.000
P Wave Duration in V1, ms [mean (SD)]	116.38 (26.06)	119.05 (32.91)	0.589
Abnormal P Wave Duration in V1 [*n* (%)]	30 (36.1)	24 (38.1)	0.945
Biphasic P Wave in V1 [*n* (%)]	65 (77.4)	51 (78.5)	1.000
Negative P Wave in V1 (*n* (%)	10 (11.9)	5 (7.7)	0.567
Bifid P Wave in V1 [*n* (%)]	3 (3.6)	2 (3.1)	1.000
Abnormal P Wave Morphology in V1 [*n* (%)]	13 (15.5)	7 (10.8)	0.553
Amplitude P Wave in V1, mV [mean (SD)]	0.05 (0.04)	0.06 (0.06)	0.061
Advanced Interatrial Block [*n* (%)]	7 (8.6)	12 (18.8)	0.123
PTFV1, mV·s [mean (SD)]	0.05 (0.05)	0.06 (0.05)	0.465
Abnormal PTFV1 [*n* (%)]	39 (49.4)	33 (57.9)	0.419
P Wave Area, mV·ms [mean (SD)]	8.40 (3.47)	8.22 (3.94)	0.766
Abnormal P Wave Area [*n* (%)]	71 (85.5)	55 (84.6)	1.000
P Wave Dispersion, ms [mean (SD)]	32.86 (19.19)	45.31 (23.05)	<0.001
Abnormal P Wave Dispersion [*n* (%)]	33 (39.3)	41 (63.1)	0.007

### 3.4 Subgroup analysis of TF patients

Among the 65 patients with confirmed thrombus or pre-thrombotic formations, 52 (80%) were classified as having prethrombotic formations, while 13 (20%) had solid thrombi in the LA. Compared to patients with prethrombotic formation, those with solid thrombus showed statistically significant differences in only a few clinical parameters. Serum creatinine levels were significantly higher in the solid thrombus group (1.37 ± 0.54 mg/dL vs. 1.06 ± 0.44 mg/dL, *p* = 0.032 and eGFR (using the CKD-EPI creatinine-based equation) was significantly lower in patients with solid thrombus (53.85 ± 20.31 mL/min/1.73 m^2^ vs. 66.72 ± 17.87 mL/min/1.73 m^2^, *p* = 0.028). A significant difference was also observed in QTc intervals, which were prolonged in the solid thrombus group (462.92 ± 41.13 ms vs. 440.87 ± 30.36 ms, *p* = 0.033). A comparison of baseline characteristics and ECG parameters between the two subgroups can be found in the (supplementary material Table S1.

### 3.5 Machine learning performance

**Figure 3:**
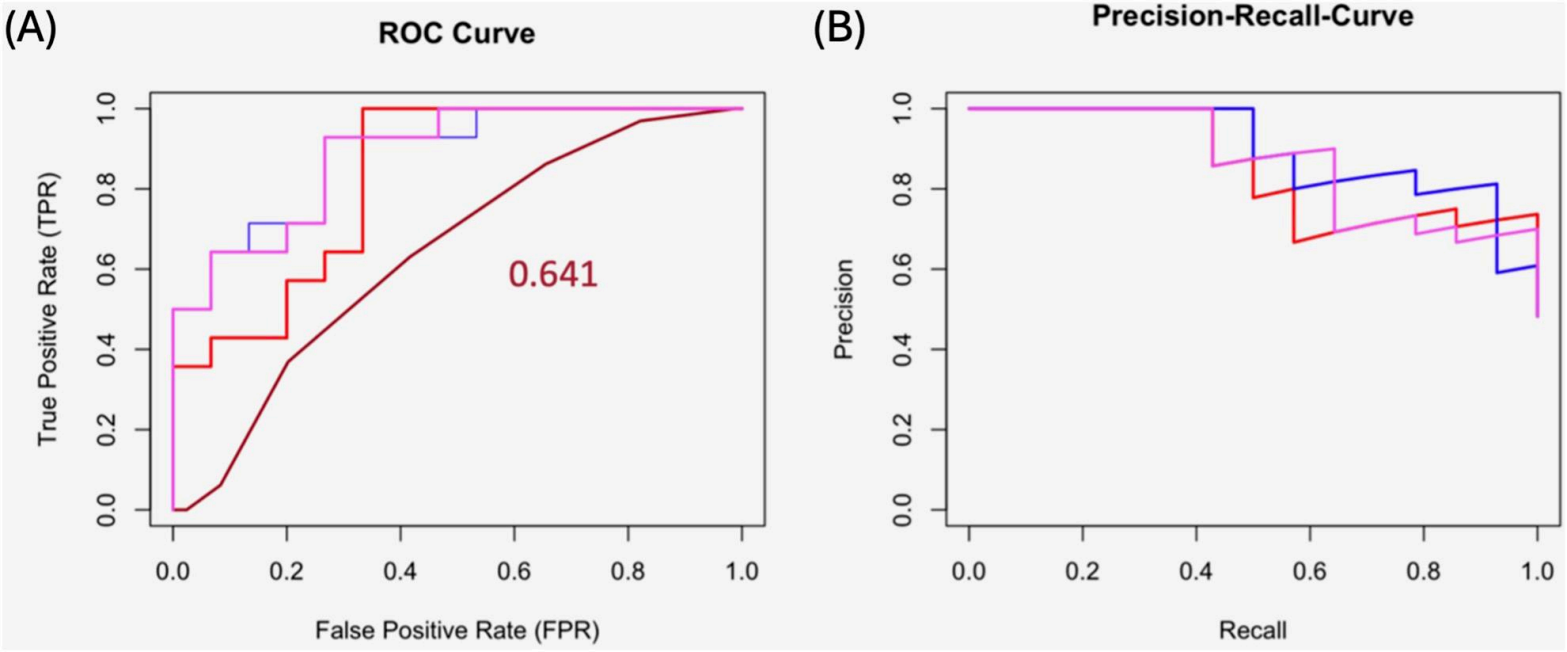
Receiver operating characteristic curve (**A**) and precision-recall-curve (**B**); XG-boost model (violet), random forest model (red), light GB model (blue), CHA_2_DS_2_-VASc score (brown).

All three ML models demonstrated relevant predictive performance in distinguishing AF patients with TF from patients without TF. All precision metrics can be found in Table 4. Both XGBoost and Random Forest yielded identical performance metrics. They achieved an overall accuracy of 82.8 percent, with a 95 percent confidence interval ranging from 64.2 to 94.1 percent. The sensitivity of both models was high at 92.9 percent, indicating a strong ability to correctly identify patients with thrombus formation. Specificity was 73.3 percent, reflecting a good balance in correctly identifying patients without thrombus. The positive predictive value (PPV) was 76.5 percent, and the negative predictive value (NPV) reached 91.7 percent. These values suggest that false positives and false negatives were both relatively low. The F1 score for both models was 0.8387, indicating a balanced performance in terms of precision and recall. The ROC AUC (see Figure 3) was 0.8857, and the PR AUC was 0.8870 for XGBoost and 0.8896 for Random Forest, indicating excellent discriminative performance. Furthermore, the McNemar's test *p*-value was 0.3711 for both models, indicating no statistically significant difference between observed and predicted classifications, which supports the robustness of these models. The LightGBM model showed slightly lower overall performance. While it achieved a perfect sensitivity of 100 percent, its specificity was only 60.0 percent. As a result, the accuracy of the LightGBM model was only 79.3 percent. Its positive predictive value was 70.0 percent, and the negative predictive value was 100 percent, consistent with its perfect sensitivity. The F1 score of LightGBM was 0.8125, slightly lower than that of the other models. The ROC AUC was 0.8286, and the PR AUC was 0.8141, indicating a modest decline in discriminative ability compared to XGBoost and Random Forest. In comparison, in this matched cohort the CHA₂DS₂-VASc Score yielded a ROC AUC of only 0.641.

**Table 4:** Precision metrics of the models.

Performance Metric	XG-Boost	Random Forest	Light GB
Optimal Cut Off	0.243	0.3884746	0.005346954
Accuracy	0.8276	0.8276	0.7931
95% CI	(0.6423, 0.9415)	(0.6423, 0.9415)	(0.6028, 0.9201)
No-Information Rate	0.5172	0.5172	0.5172
*P*-Value [Acc > NIR]	0.0005252	0.0005252	0.002088
Kappa	0.6572	0.6572	0.5915
Mcnemar's Test *P*-Value	0.3710934	0.3710934	0.041227
Sensitivity	0.9286	0.9286	1.0000
Specificity	0.7333	0.7333	0.6000
Pos Pred Value	0.7647	0.7647	0.7000
Neg Pred Value	0.9167	0.9167	1.0000
Prevalence	0.4828	0.4828	0.4828
Detection Rate	0.4483	0.4483	0.4828
Detection Prevalence	0.5862	0.5862	0.6897
Balanced Accuracy	0.8310	0.8310	0.8000
F1 Score	0.8387	0.8387	0.8125
Cross Validation Mean AUC	0.7307		0.7356
ROC AUC	0.8857	0.8857	0.8286

AUC, area under the curve; ROC, receiver operating characteristic; PR, precision-recall; NIR, no-information rate; CV, cross-validation.

### 3.6 Feature importance analysis

**Figure 4:**
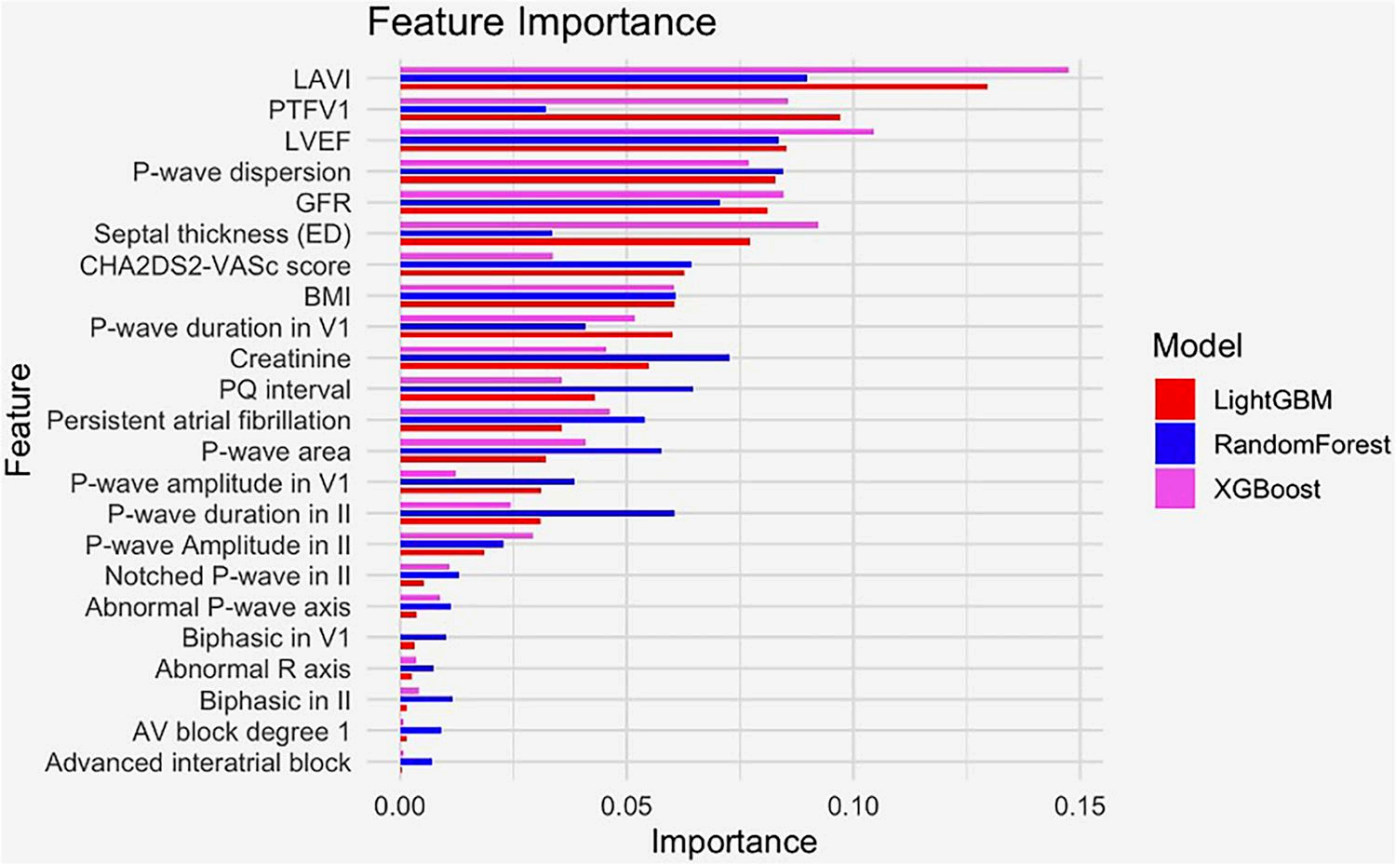
P-wave morphology parameters for atrial cardiomyopathy in TF+ and TF− patients. Panels a-i display boxplots comparing quantitative P-wave parameters between patients with (TF+, dark blue) and without thrombus (TF−, light blue), Panel j shows the distribution of patients by number of abnormal P-wave parameters within each group. Panel k displays the proportions of patients in each group presenting with specific categorical p-wave morphology parameters. (Biphasic in V1, Biphasic P Wave Morphology in V1, Negative in V1, Negative P-Wave in V1).

**Figure 5:**
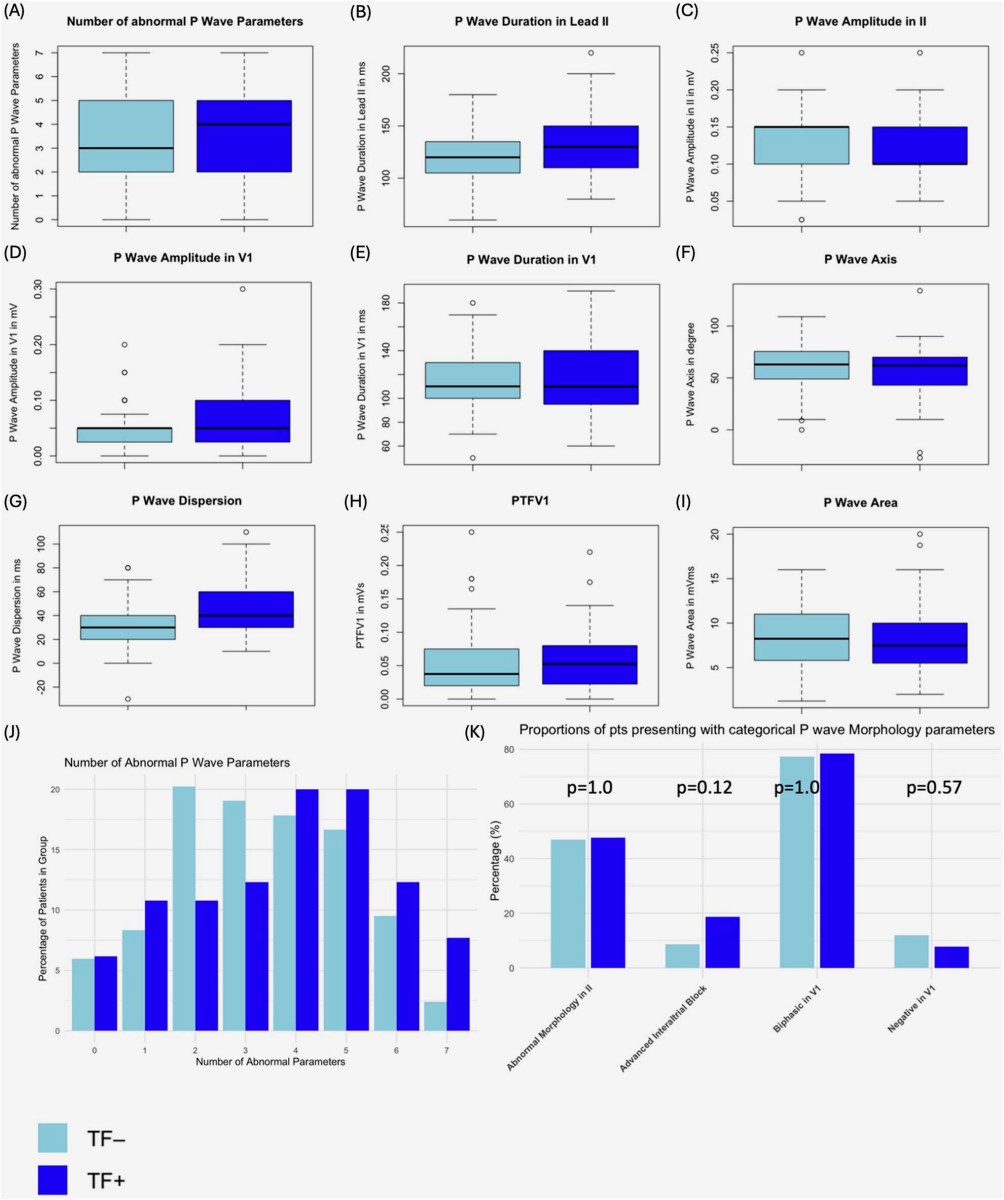
Feature importance of the ML models; (**A**) Number of abnormal P Wave Parameters; (**B**) P Wave Duration in Lead II; (**C**) P Wave Amplitude in II; (**D**) P Wave Amplitude in V1; (**E**) P Wave Duration in V1; (**F**) P Wave Axis; (**G**) P Wave Dispersion; (**H**) PTFV1; (**I**) P Wave Area; (**J**) Number of Abnormal P Wave Parameters; (**K**) Proportions of patients presenting with categorial P Wave Morphology parameters.

The relative contribution of individual features to thrombus prediction was assessed across all three ML models. All Model Features and the importance each can be found in Figures 4, 5.

Left atrial volume index (LAVI) consistently emerged as the most important predictor in all models. P terminal force in lead V1 (PTFV1), left ventricular ejection fraction (LVEF), P-wave dispersion, and glomerular filtration rate (eGFR) were also among the top-ranked features across models.

Other relevant predictors included septal thickness in end-diastole, CHA₂DS₂-VASc Score, and P-wave duration in lead V1. Notably, the importance of ECG-derived atrial conduction parameters such as P-wave duration and amplitude, P-wave axis abnormalities, and biphasic P-waves varied between models but contributed consistently to model performance. While the specific ranking of features differed slightly between the three algorithms there is a significant overlap in key predictors. underscores the potential clinical value of combining echocardiographic, electrophysiological, and laboratory parameters for risk stratification.

**Figure 6:**
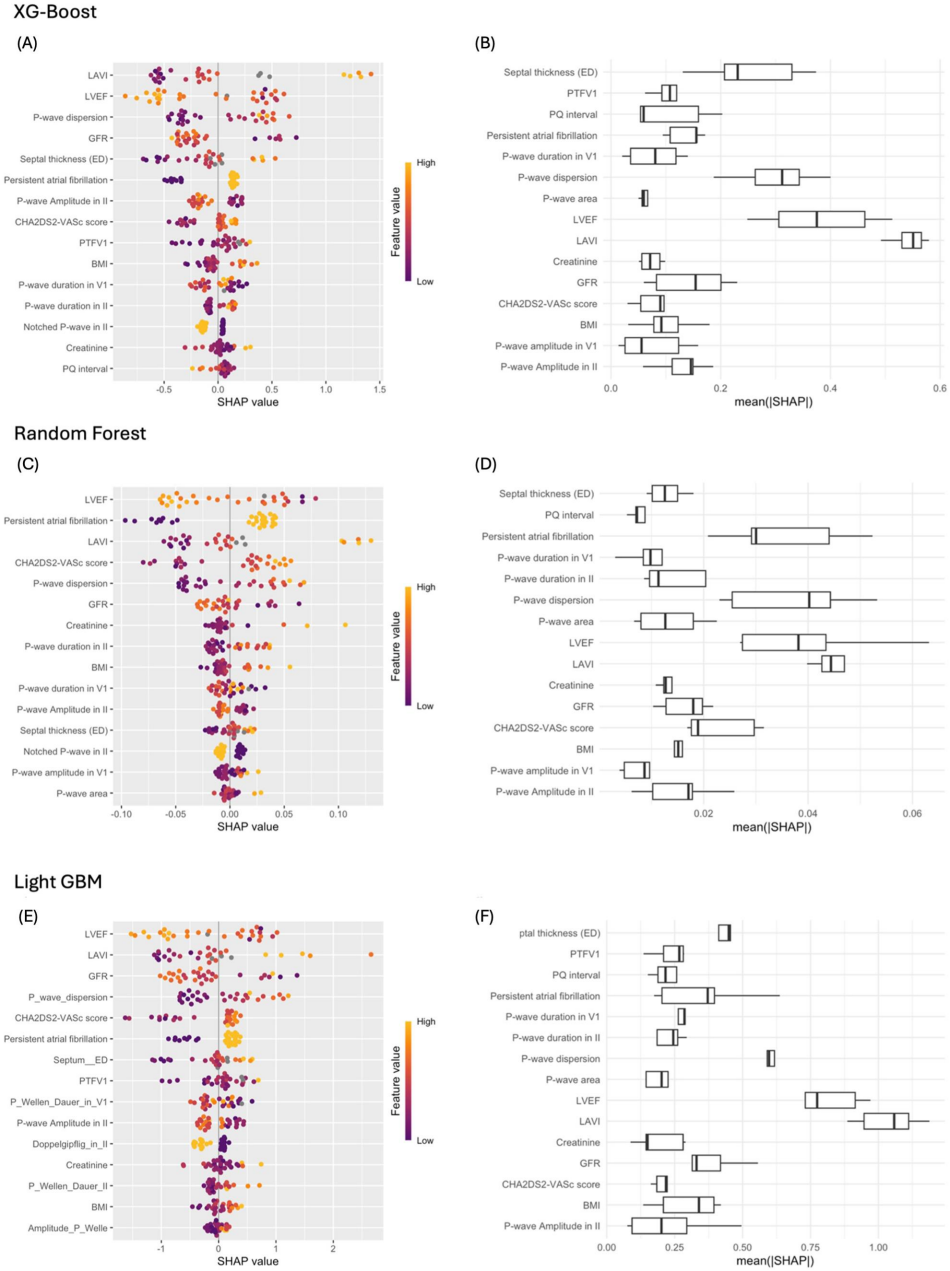
SHAP-based feature importance of the 15 most relevant predictors in the three machine learning models. Panels **(A)**, **(C)**, and **(E)** show SHAP beeswarm plots for XGBoost, Random Forest, and LightGBM. Each dot represents an individual patient, with color indicating the relative feature value (purple = low, yellow = high). Panels **(B)**, **(D)**, and **(F)** display boxplots of the mean absolute SHAP values across cross-validation folds.

The shap analysis (see Figure 6) supports the thesis that all three models consistently use a similar set of clinically predictors. The beeswarm panels demonstrate the direction and dispersion of effects at the patient level: higher LVEF and smaller eGFR shift predictions downward, whereas larger LAVI and P-wave dispersion tend to shift them upward. The present of persistent atrial fibrillation, increases the predictive value.

The fold-wise boxplots of mean absolute SHAP values show that the median importances for the leading features are stable with relatively narrow interquartile ranges, supporting robustness under resampling.

The three to four most influential features in each model clearly stand apart from the others, even though the ranking may vary slightly due to overlap in the interquartile ranges. P-wave dispersion is under those relevant features in every model, underling its importance for the prediction.

### 3.7 External validation

All three models discriminated between patients with and without TF in the external validation cohort (Table 5). Across models, the classification thresholds selected in the external validation cohort were higher than the thresholds selected in the internal validation. When applying the lower, training-derived thresholds to the external cohort, the models achieved 100% sensitivity (no false negatives) and produced a higher number of false positives. In the external validation cohort, the random forest yielded the highest performance metrics among the three models, with only small differences between models.

**Table 5:** Results from the external validation cohort.

Performance Metric	XG-Boost	Random Forest	Light GBM
Prior Chosen Cut-Off Value	0.4	0.5	0.2
Sensitivity	1.0000	1.0000	1.0000
Specificity	0.4286	0.7143	0.5714
True Positive	2	2	2
True Negative	6	10	8
False Positive	8	4	6
False Negative	0	0	0
Optimal Cut-Off Value	0.928	0.62	0.9995
Sensitivity	1.0000	1.0000	1.0000
Specificity	1.0000	0.9286	0.9286

ECG, electrocardiogram; QTc, corrected QT interval; LBBB, left bundle branch block; RBBB, right bundle branch block; PTFV1, P-terminal force in lead V1.

## 4 Discussion

Despite continuous OAC, left atrial thrombus risk persists in a relevant subset of patients.

Our observational study demonstrated the following:

- Thrombus formations can be detected by TOE in patients undergoing CA procedures, despite continuous OAC and a high prevalence of DOAC use—even occurring in patients with low-moderate thromboembolic risk scores
- Our three multimodal ML models, which integrate clinical, echocardiographic, and electrocardiographic features of SR ECGs demonstrated a high predictive performance for thrombus formation and performed better than traditional CHA₂DS₂-VASc Scoring in our cohort

### 4.1 Preprocedural TOE imaging and stroke risk score recommendations

Atrial thrombi can occur in a subset of patients in patients undergoing cardioversion or ablation. A meta-analysis of 35 studies (*n* = 14,635) reported a 2.7% prevalence prior cardioversion or CA under continuous OAC, while single-centre and registry studies have shown higher rates of 7.5−7.9%, independent of OAC type (3, 23). In our study, the incidence was 6.96% in patients undergoing CA procedures, which is within this range; however, our analysis included both thrombi and pre-thrombotic formations eluding CA. Of note, the clinical utilization of TOE prior to catheter ablation or cardioversion for atrial fibrillation (AF) is decreasing from 86% in 2010 (2) to 15−59% (2, 24–26) in the last years likely due to increased confidence in uninterrupted anticoagulation protocols. In our study, by the uniform use of TOE for all eligible patients confounding by indication and therefore/work-up bias was minimized. Although Current guidelines recommend individualized preprocedural decision-making, primarily guided by the CHA₂DS₂-VASc score and anticoagulation status our model relies on pre-imaging data and is designed to triage patients to TOE/CT as imaging rates decline, supporting safe, targeted use in high-risk individuals rather than routine imaging of all patients. The latest expert consensus statement on CA of AF highlights preprocedural imaging for thrombus exclusion in patients at increased risk (CHA₂DS₂-VASc ≥3) as an area of uncertainty. It suggests that TOE may be reasonable in these patients, even after at least three weeks of therapeutic OAC (2). This recommendation is supported by data showing a significantly higher prevalence of LA thrombus in patients with CHA₂DS₂-VASc Scores ≥3 compared to those with scores ≤2 (6.3% vs. 1.1%, *p* < 0.001) (3). In our study, TF+ patients had higher CHA₂DS₂-VA and CHA₂DS₂-VASc Scores compared to TF− patients. Still, 13.9% of our TF+ patients had a CHA₂DS₂-VASc Score ≤ 2 and therefore, might have not undergone TOE according to those recommendations and some centers do not even base their decision making for TOE on CHA₂DS₂-VA(Sc) Score when patients are adequately anticoagulated. Consequently, some patients may undergo unnecessary TOE procedures, while others may face an underestimated risk, potentially leading to peri-procedural thromboembolic complications. Of note, the diagnostic performance of our ML models (ROC AUC 0.886, 0.886 and 0.826) was significantly better than that of the CHA₂DS₂-VASc Score alone in our population (ROC AUC 0.641). These results support the use of ensemble-based ML methods for TF risk classification.

Therefore, a stroke risk score approach may not fully capture individual thromboembolic risks, particularly in patients with intermediate risk as these scores do not account for the underlying atrial substrate. ACM is a major contributor to atrial thrombogenicity and remains unrepresented in current scoring systems and is, in fact, challenging to detect.

Cardiac MRI studies reveal that approximately 25% of AF patients with a low CHA₂DS₂-VASc Score (0 or 1) already exhibit significant left atrial fibrosis (UTAH stages II-IV), a known substrate for thrombus formation. However, despite its utility in detecting atrial fibrosis, routine use of cardiac MRI remains limited due to technical challenges, lack of standardization, costs and availability (26, 27).

### 4.2 AF burden

AF Burden plays a significant role for thrombogenesis with persistent AF patients having a 4−6 fold higher likelihood of LA thrombi compared to paroxysmal AF patients (3, 28).

In our cohort 96.9% of our TF+ patients were in atrial arrhythmias at the time of TOE (AF 89.2%, AT 4.7%, typical flutter 1.5%) and those 2 patients (3.1%) who presented in SR have had an electric cardioversion within 1 week before thrombus detection. Therefore, the duration of the current clinical arrhythmia may be added to guide pre-interventional imaging decisions. Interestingly, persistent AF was not ranked in the top 10 of our ML features, which might be due to the fact that the percentage of persistent AF patients (71.1%) was high in our population, even though there was a significant difference between the TF+ (87,7%) and the TF− (58.3%) group (*p* < 0.001).

### 4.3 Clinical parameters and TF risk

Some clinical parameters from the electronic health records (EHR) seem to be of special importance for thrombus prediction based on our ML models. LAVI and LV-EF rank as important features in all three models, while CHA₂DS₂-VASc Score is a top 10 feature in only 2 of 3 models. Left atrial enlargement (≥50 mm), and reduced ejection fraction (<50%) have been identified as independent risk factors before (28–30). The prevalence of TF in patients with LV-EF <40% was shown to be 22% in the LATTEE registry and a cutoff value of <48% was established which accurately detected TF (AUC 0.74) (30). Mechanistically, LV systolic dysfunction elevates atrial pressures, induces structural remodeling and fibrosis characteristic of ACM, and precipitates stasis in the left atrial appendage.

### 4.4 Role of SR ECG parameters for detecting atrial cardiomyopathy and TF risk

We might need more non-invasive and widely accessible diagnostic tools, such as (SR) ECGs in addition to clinical parameters to assess the progression of AF and enhance AF phenotyping concerning thrombus risk under OAC. ACM encompasses structural, functional, and electrophysiological alterations of the atria, which also manifest as distinct ECG abnormalities during SR. It was shown that ACM can manifest as prolonged amplified P-duration being significantly longer in patients with LA thrombus than in controls. P-wave duration and other P-wave indices, including P-wave dispersion and PTFV1, were shown to be closely associated with atrial fibrosis and low-voltage areas on electroanatomical mapping with the latter being a strong predictor of TF (31).

A recent ML model integrated preprocedural, non-invasive SR ECG parameters—such as P-wave duration and PR interval combined with intraprocedural conduction data from intracardiac electrograms obtained during electrophysiological studies improving the prediction of arrhythmia recurrence following CA which is also a sign of atrial substrate progression (32).

In our study 3−4 pathological P-wave characteristics in SR ECGs were stated underneath the 10 most important features of all 3 ML models. P-wave dispersion and PTFV1 were the most important P-wave features in XG-Boost and LightGB, in the Random Forest model P-wave dispersion and P-wave area had the highest impact. P-wave duration and P-wave dispersion were previously shown to predict postablation AF recurrences (33) as a surrogate of atrial substrate presence. P-wave abnormalities in SR ECGs were observed in a substantial proportion of our study patients. 72.3% showed at least 3 pathological P-wave-morphology features. Moreover, 41.5% of the TF+ patients have had ≥1 CA procedure at the time of thrombus detection and the TF+ group was older compared to typical CA populations representing a population with an advanced atrial disease.

The use of ECG data provides a practical and efficient means of capturing atrial substrate abnormalities, potentially obviating the need for advanced imaging modalities such as cardiac MRI or time-intensive diagnostic assessments like atrial strain or flow measurements, which are often limited in routine clinical practice.

Complementing this, groundbreaking work by the Mayo Clinic group (34, 35) has demonstrated that AI enabled analysis of SR ECGs can accurately predict incident AF well before clinical manifestation, by capturing subtle electrophysiological signs of atrial remodeling. This paradigm opens the door for implementing ECG-based algorithms into longitudinal EHR systems, enabling dynamic, real-time risk evaluation for incident AF (currently already implemented in some centers) but maybe, in the future, even estimate progression of AF/ACM and therefore predict stroke or LA thrombus risk throughout a patient's lifetime.

Ultimately, combining ECG-derived features with clinical, imaging, biomarker and AI-driven analytics holds promise for a transformative shift in AF management beyond traditional scores.

### 4.5 Role of machine leaning in phenotyping AF patients

Adding ML to our traditional risk scores seems promising for TF prediction.

A ML approach used on the LATTEE registry data found that using TTE and clinical data outperformed classic risk stratification models such as LV-EF and CHA₂DS₂-VASc Score (36). In difference to our data a mixed electric cardioversion/AF ablation dataset was used without implementing of ECG data. Our data showed a significant overlap in key predictors for TF, which underscores the potential clinical value of multimodal ML models combining clinical EHR data, echocardiographic, electrophysiological (like ECG markers for ACM) and laboratory parameters for risk stratification, especially when larger datasets can be analyzed.

New and established circulating biomarkers—including age-adjusted D-dimer, natriuretic peptides (BNP/NT-proBNP), high-sensitivity C-reactive protein, von Willebrand factor, and fibrin-clot phenotype—have each been linked to left-atrial(appendage) thrombus or a prothrombotic atrial milieu in AF (37–40), so they merit consideration as features in future thrombus-prediction models. For example, Almorad et al. showed that age-adjusted D-dimer (10× age) achieved 100% sensitivity and negative predictive value to rule out left atrial thrombus and would have safely obviated pre-cardioversion TOE in ∼61% of AF patients, supporting its use as a simple triage tool (37).

The type or intensity of OAC was not included as a predictor in our model. Therefore, in clinical practice risk estimates are not a proxy for treatment allocation. High-risk outputs could prompt optimization of guideline-concordant OAC (adherence, on-label dosing, drug–drug interactions) and targeted pre-procedural LAA imaging (TEE/CT), not anticoagulation escalation or ablation deferral without confirmed thrombus. Using the prespecified cut-offs (Table 4), the model supports targeted imaging of model-positive patients. For clinical routine a pre-assessment 4 weeks before ablation and a day-of-procedure re-check to incorporate updated data could be introduced. Overall, models like ours might function as a triage tool that prioritizes imaging and reinforces guideline-concordant care while avoiding unnecessary imaging and delays in low-risk patients.

Therefore, with ML tools the clinical gap between guideline recommendations and real-world practice concerning the use of TOE in patients undergoing CA or cardioversion for AF might be closed soon.

### 4.6 Limitations

This study has several limitations inherent to its design and methodology. First, despite the inclusion of an external validation cohort from a separate institution, the primary dataset was derived from a single center, which may limit the generalizability of the findings and introduces the inherent risk of selection bias, incomplete data capture, and information bias related to chart-based data extraction. These limitations are intrinsic to retrospective database studies and support the exploratory character of the present analysis. Our hypothesis-generating pilot study requires multi-center validation in larger cohorts before any clinical application. Second, the relatively small sample size contains the robustness of the ML model. This is mainly since we focused on AF ablation patients with continuous OAC and excluded patients without previous SR ECGs from the analysis (most of the patients were in persistent AF). Although performance metrics remained consistent across training and validation subsets, small datasets carry an increased risk of model overfitting and may not fully capture the heterogeneity of the AF population. This limitation is well recognized in prior ML-based cardiovascular research, where sample size and data diversity are critical for model reliability and clinical applicability (34, 41). Furthermore, while we applied state-of-the-art techniques such as cross-validation and propensity matching to minimize bias, we acknowledge that small, imbalanced datasets can still lead to optimistic performance estimates. In addition, the final matched study cohort represented only a small fraction of the initially available population. Inclusion of all remaining patients into the analysis was not feasible, as manual review of medical charts, ECGs, and echocardiographic reports was required, and regional regulatory restrictions currently do not allow automated extraction of electronic health record data for research purposes in the necessary extent. LLM-based cohort builder that can perform automated extraction from pseudonymized medical records might help in the future. The substantial reduction in sample size may have excluded potentially informative clinical characteristics and limits the representativeness of the analysed cohort. The need for available sinus rhythm ECGs may have introduced selection bias and reduced generalizability. Thus, the present model was developed in a highly selected population and may not fully reflect the broader spectrum of anticoagulated AF patients encountered in routine practice. Moreover, although the test set was defined according to a conventional 20% split, the absolute number of thrombus cases in the test cohort remained very small. Consequently, test performance estimates must be interpreted with caution, as they are statistically unstable and cannot provide robust evidence for generalizability or clinical usefulness.

Moreover, propensity score matching is not a standard approach in Machine leaning but was used in other studys before (42). Since age is one of the most powerful determinants of thromboembolic stroke in AF and the role of sex is a well-known risk modifier (43, 44), we aimed to minimize the influence of those parameters in order to identify additional, potentially relevant predictors for TF and to create a more challenging and conservative modeling setting. However, this approach may also have attenuated the contribution of two clinically relevant determinant and may limit the external validity and needs to be testes in bigger data sets.

The external validation cohort was also very small and therefore provides only limited support for model transferability. In particular, the modest specificity observed in this cohort underlines that the model is not yet sufficiently robust for routine clinical application, but should be regarded as a exploratory pilot study. The cut-off value for classification could not be reliably determined due to the small sample size of the training and validation cohorts. The observed need to adjust the threshold in the external validation indicates that the model, in its current form, is not yet suitable for direct clinical implementation. Applying training-derived thresholds resulted in maximal sensitivity at the expense of more false positives, which may be an acceptable trade-off for a screening-oriented use case where avoiding missed TF is prioritized; however, prospective evaluation is required to determine the most appropriate operating point and to confirm generalizability across broader populations. Larger training datasets will be required in future studies to establish a robust and generalizable cut-off value (42). Also, systematic hyperparameter tuning with a dedicated search strategy might further improve performance and has not yet been applied. Furthermore, the clinical significance of prethrombotic formations remains uncertain, and data comparing the stroke risk of such formations—particularly sludge—to that of solid thrombi are lacking. Nevertheless, it is standard clinical practice to defer electrical cardioversion or CA when prethrombotic formations are present. However, a thrombus does not necessarily represent an actual stroke risk. A multicenter study of 1,155 patients undergoing CA reported a very low thromboembolic event rate (0.35%), despite preprocedural TOE being performed in only 22.6% of cases even though just 56% of those patients were in AF at the time of presentation, indicating a lower AF burden and possibly lower risk compared to our population (45). Another limitation is that we were not able to include echocardiographic data on left atrial function into our model (like strain analysis) or even use unprocessed imaging data which might have further enhanced ML model performance. Future work should aim to validate the model prospectively in larger, multicentric cohorts and assess its clinical utility across varying patient populations.

### 4.7 Clinical utility

The clinical applicability of the present model is currently limited. The reported performance metrics were derived from a retrospectively assembled, single-center, matched cohort with an artificially increased prevalence of thrombus formation and therefore cannot be directly transferred to routine clinical decision-making in broader anticoagulated AF populations with substantially lower event rates. Moreover, the external validation cohort was very small, limiting conclusions regarding generalizability. Accordingly, this model should be interpreted as a proof-of-concept approach requiring extensive validation before any clinical implementation can be considered.

## 5 Conclusion

In our study we demonstrated that ML algorithms offer a promising tool for predicting TF in AF patients with continuous OAC undergoing CA using existing medical records. The combination of clinical data with echocardiographic and electrocardiographic parameters suggesting an atrial substrate showed a high predictive accuracy. An AI-based protocol may support clinical decision-making regarding the necessity of TOE in chronically anticoagulated patients scheduled for CA and may further reduce periprocedural stroke risk in the future.

## Data Availability

The raw data supporting the conclusions of this article will be made available by the authors, without undue reservation.
