# Supplementary material for: Intracardiac thrombus formations despite continuous oral anticoagulation in atrial fibrillation patients undergoing catheter ablation procedures: pilot development of a machine learning prediction model
Source: Front Cardiovasc Med. 2026 Jun 4;13:1707003. doi: 10.3389/fcvm.2026.1707003 (PMC13275220; doi:10.3389/fcvm.2026.1707003)

Supplementary Material

# Supplementary Data

*Table S1: Baseline Characteristics and Sinus Rhythm ECG Parameters compared between Patients with a prethrombotic formation and Patients with a solid LA thrombus.
Abbreviations: TF = thrombus formation; BMI = body mass index; AF = atrial fibrillation; TIA = transient ischemic attack; GFR = glomerular filtration rate; LAVI = left atrial volume index; LVEF = left ventricular ejection fraction; ED = end-diastole; ECG = electrocardiogram; QTc = corrected QT interval; LBBB = left bundle branch block; RBBB = right bundle branch block; PTFV1 = P-terminal force in lead V1.*

|  | Prethrombotic Formation | Solid LA or LAA Thrombus | p |
| --- | --- | --- | --- |
| n | 52 | 13 |  |
| Baseline Characteristics |  |  |  |
| BMI (mean (SD)) | 28.19 (5.53) | 28.84 (5.51) | 0.704 |
| Persistent AF (n (%)) | 46 (88.5) | 11 (84.6) | 1.000 |
| Sex (n (%)) | 20 (38.5) | 8 (61.5) | 0.234 |
| Structural Heart Disease (n (%)) | 22 (42.3) | 6 (46.2) | 1.000 |
| Renal insufficiency (n (%)) | 16 (30.8) | 7 (53.8) | 0.218 |
| Congestive Heart failure (n (%)) | 27 (51.9) | 11 (84.6) | 0.068 |
| Hypertension (n (%)) | 49 (94.2) | 12 (92.3) | 1.000 |
| Diabetes (n (%)) | 16 (30.8) | 4 (30.8) | 1.000 |
| TIA (n (%)) | 1 ( 1.9) | 0 ( 0.0) | 1.000 |
| Stroke (n (%)) | 6 (11.5) | 2 (15.4) | 1.000 |
| Vascular Disease (n (%)) | 27 (51.9) | 5 (38.5) | 0.577 |
| Age (mean (SD)) | 71.54 (7.85) | 69.54 (9.49) | 0.434 |
| CHA₂DS₂-VA (n (%) |  |  | 0.545 |
| 1 | 2 ( 3.8) | 0 ( 0.0) |  |
| 2 | 6 (11.5) | 1 ( 7.7) |  |
| 3 | 11 (21.2) | 4 (30.8) |  |
| 4 | 15 (28.8) | 2 (15.4) |  |
| 5 | 14 (26.9) | 6 (46.2) |  |
| 6 | 4 ( 7.7) | 0 ( 0.0) |  |
| Creatinine, mg/dL (mean (SD)) | 1.06 (0.44) | 1.37 (0.54) | 0.032 |
| GFR, mL/min/1.73 m² (mean (SD)) | 66.72 (17.87) | 53.85 (20.31) | 0.028 |
| LAVI, mL/m² (mean (SD)) | 49.78 (12.98) | 56.77 (17.10) | 0.116 |
| LVEF, % (mean (SD)) | 52.04 (10.73) | 46.85 (13.13) | 0.144 |
| Septum Thickness, ED, mm (mean (SD)) | 12.11 (2.00) | 11.75 (1.96) | 0.580 |
| Posterior Wall, ED, mm (mean (SD)) | 10.24 (1.89) | 10.36 (1.63) | 0.850 |
| ECG Parameters |  |  |  |
| PQ-Time, ms (mean (SD)) | 191.04 (37.44) | 201.23 (49.23) | 0.414 |
| QRS-Duration, ms (mean (SD)) | 102.40 (19.76) | 111.62 (24.91) | 0.159 |
| QT-Time, ms (mean (SD)) | 424.21 (37.78) | 436.31 (61.75) | 0.372 |
| QTc, ms (mean (SD)) | 440.87 (30.36) | 462.92 (41.13) | 0.033 |
| P-Axis, degree (mean (SD)) | 57.92 (21.32) | 50.85 (41.50) | 0.390 |
| R-Axis, degree (mean (SD)) | 27.17 (44.55) | 14.00 (51.60) | 0.359 |
| AV-Block degree 1 (n (%)) | 16 (30.8) | 6 (46.2) | 0.471 |
| Bundle Branch Block (n (%)) |  |  | 0.461 |
| None | 39 (75.0) | 9 (69.2) |  |
| LBBB | 5 ( 9.6) | 3 (23.1) |  |
| RBBB | 4 ( 7.7) | 0 ( 0.0) |  |
| Incomplete RBBB | 4 ( 7.7) | 1 ( 7.7) |  |
| P-Wave Duration in II, ms (mean (SD)) | 132.69 (30.80) | 126.92 (24.96) | 0.534 |
| Biphasic in II (n (%)) | 13 (25.0) | 3 (23.1) | 1.000 |
| Bifid in II (n (%)) | 11 (21.2) | 4 (30.8) | 0.713 |
| Amplitude P-Wave in II, mV (mean (SD)) | 119.20 (32.63) | 118.46 (35.32) | 0.943 |
| P-Wave Duration in V1, ms (mean (SD)) | 0.12 (0.05) | 0.12 (0.05) | 0.543 |
| Biphasic in V1 (n (%)) | 41 (78.8) | 10 (76.9) | 1.000 |
| Negative in V1 (n (%)) | 4 ( 7.7) | 1 ( 7.7) | 1.000 |
| Bifid in V1 (n (%)) | 2 ( 3.8) | 0 ( 0.0) | 1.000 |
| Amplitude P-Wave in V1, mV (mean (SD)) | 0.07 (0.06) | 0.05 (0.04) | 0.412 |
| Advanced Interatrial Block (n (%)) | 11 (21.2) | 1 ( 8.3) | 0.538 |
| PTFV1, mV·s (mean (SD)) | 0.06 (0.04) | 0.07 (0.05) | 0.509 |
| P-wave area, mV·ms (mean (SD)) | 8.39 (4.01) | 7.52 (3.73) | 0.481 |
| P-wave dispersion, ms (mean (SD)) | 46.25 (22.75) | 41.54 (24.78) | 0.514 |

*Table S2: Baseline Characteristics compared between the Groups before and after exclusion of the patients without SR ECG
Abbreviations: TF = thrombus formation; BMI = body mass index; AF = atrial fibrillation; CM = cardiomyopathy; TIA = transient ischemic attack; GFR = glomerular filtration rate; LAVI = left atrial volume index; LVEF = left ventricular ejection fraction; ED = end-diastole; PTT = partial thromboplastin time.*

|  | TF- (with SR ECG) | TF- before exclusion | TF+ (with SR ECG only) | TF+ before exclusion |
| --- | --- | --- | --- | --- |
| n | 84 | 93 | 65 | 93 |
| BMI (mean (SD)) | 27.10 (4.72) | 27.50 (5.55) | 28.32 (5.49) | 28.46 (5.59) |
| Persistent AF (n (%)) | 49 (58.3) | 55 (59.1) | 57 (87.7) | 85 (91.4) |
| Sex = male (n (%)) | 37 (44.0) | 44 (47.3) | 28 (43.1) | 43 (46.2) |
| Structural Heart Disease (n (%)) | 11 (13.1) | 13 (14.0) | 28 (43.1) | 38 (40.9) |
| Renal Insufficiency (n (%)) | 14 (16.7) | 17 (18.3) | 23 (35.4) | 29 (31.2) |
| Congestive Heart Failure (n (%)) | 28 (33.3) | 31 (33.3) | 38 (58.5) | 55 (59.1) |
| Hypertension (n (%)) | 64 (76.2) | 69 (74.2) | 61 (93.8) | 86 (92.5) |
| Diabetes (n (%)) | 12 (14.3) | 12 (12.9) | 20 (30.8) | 28 (30.1) |
| TIA (n (%)) | 2 ( 2.4) | 2 (2.2) | 1 ( 1.5) | 4 (4.3) |
| Stroke (n (%)) | 12 (14.3) | 14 (15.1) | 8 (12.3) | 10 (10.8) |
| Other Thromboembolic Events (n (%)) | 1 ( 1.2) | 1 (1.1) | 1 ( 1.5) | 2 (2.2) |
| Vascular Disease (n (%)) | 27 (32.1) | 32 (34.4) | 32 (49.2) | 43 (46.2) |
| Age (mean (SD)) | 72.14 (8.74) | 71.26 (7.89) | 71.14 (8.16) | 71.08 (7.94) |
| CHA₂DS₂-VASc Score (mean (SD)) | 3.19 (1.59) | 2.75 (1.46) | 3.89 (1.23) | 3.39 (1.28) |
| Creatinine, mg/dL (mean (SD)) | 0.99 (0.26) | 0.99 (0.22) | 1.12 (0.48) | 1.14 (0.48) |
| GFR, mL/min/1.73 m² (mean (SD)) | 68.42 (15.47) | 68.63 (15.60) | 64.06 (18.97) | 63.33 (19.22) |
| LAVI, mL/m² (mean (SD)) | 40.81 (13.09) | 43.62 (14.49) | 51.32 (14.13) | 55.34 (22.77) |
| LVEF, % (mean (SD)) | 56.74 (9.44) | 56.68 (9.35) | 50.95 (11.36) | 50.15 (11.63) |
| Septum Thickness, ED, mm (mean (SD)) | 10.94 (1.84) | 10.97 (1.77) | 12.04 (1.98) | 11.94 (2.03) |
| Posterior Wall, ED, mm (mean (SD)) | 9.86 (1.83) | 9.77 (1.80) | 10.27 (1.82) | 1.86) |

*Figure S1: Stability of feature rankings across cross-validation folds. Heatmaps show pairwise Spearman rank correlations between the feature rankings obtained in each of the five CV folds, computed from mean absolute SHAP values. Panels display (a) XGBoost, (b) Random Forest, and (c) LightGBM; Cell values are Spearman rank correlation coefficients; darker shading indicates stronger agreement. The diagonal equals 1.0 by definition.*

*
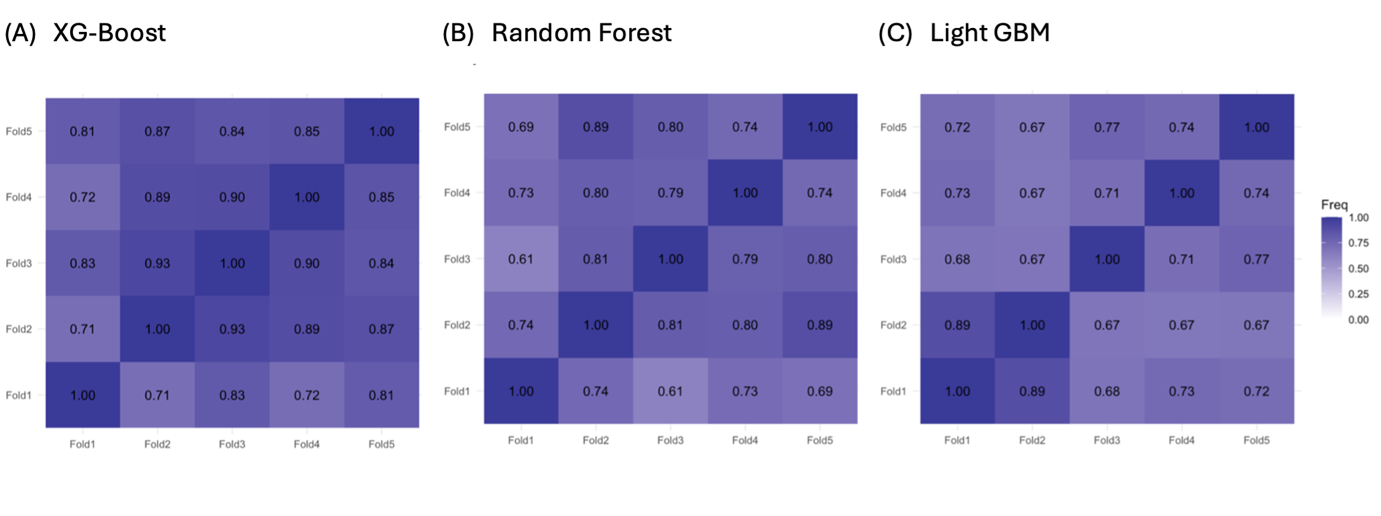
*

*Figure S2: Feature importance across models using two complementary approaches.*

*Left panels [(A), (C), (E)] show permutation importance, expressed as the AUC change when each feature is permuted; points are mean effects (over repeated permutations) and whiskers indicate 95% confidence intervals. Right panels [(B), (D), (F)] show global SHAP importance on the test set (mean absolute SHAP) with 95% bootstrap confidence intervals across observations. Features are ordered by importance within each model; only the top 15 features are displayed.*


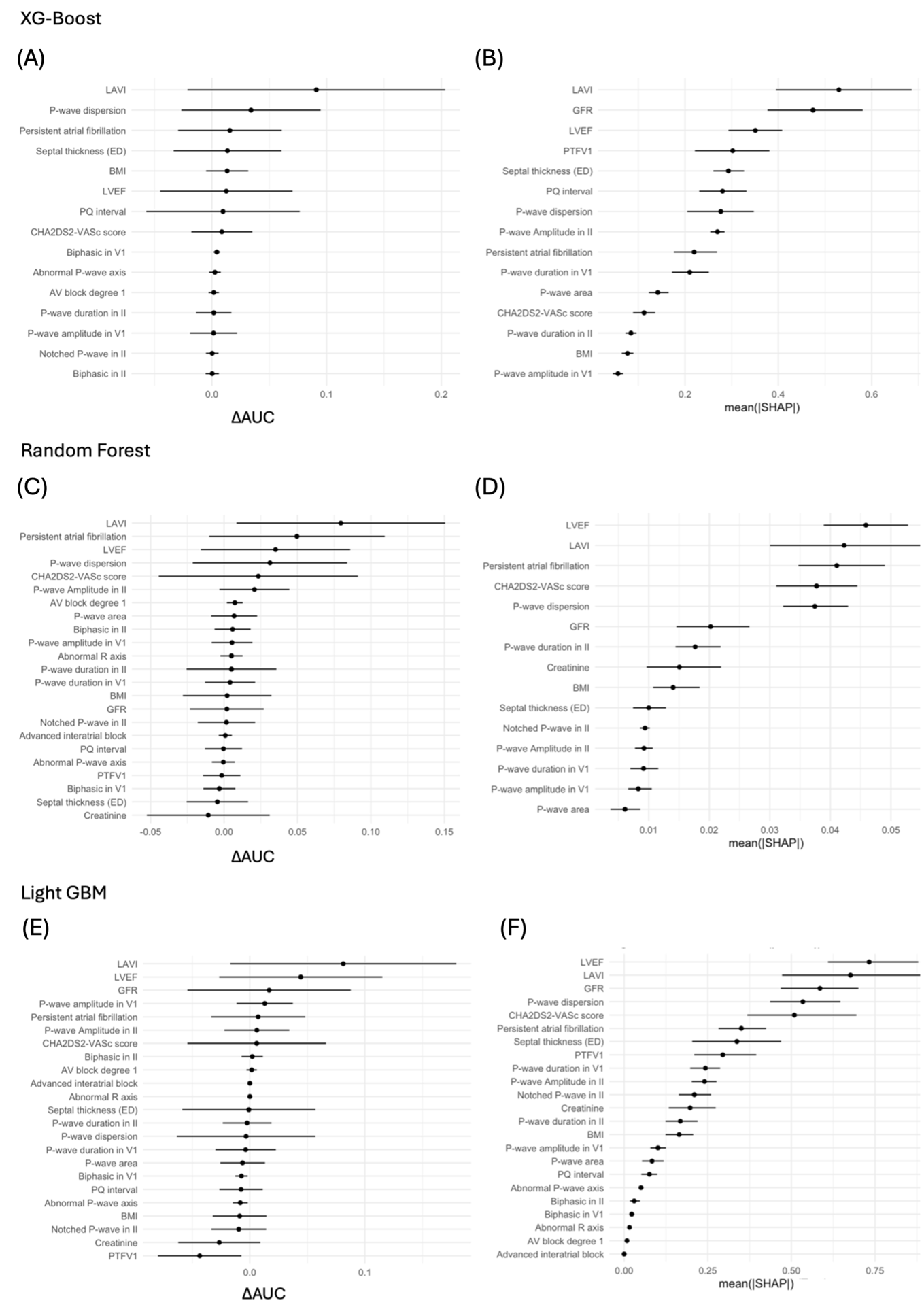

Supplement: Supplementary file 1 [file Datasheet1.docx]
